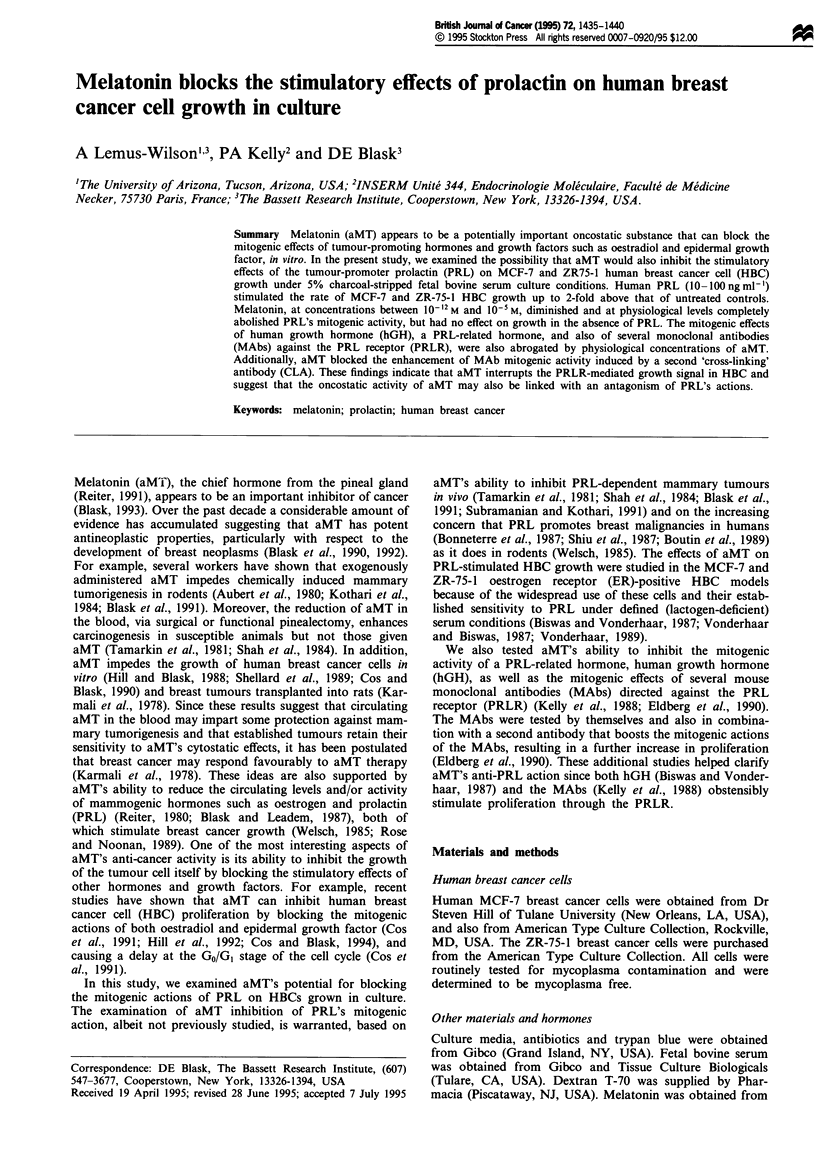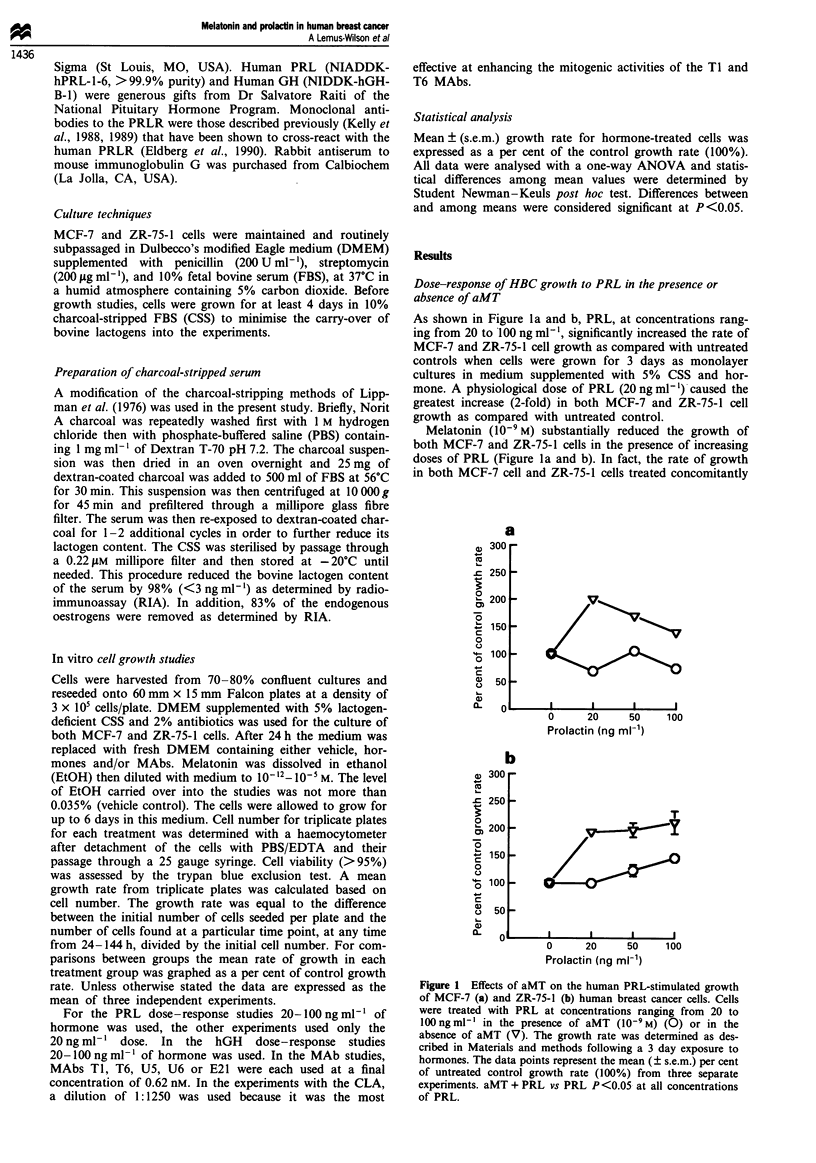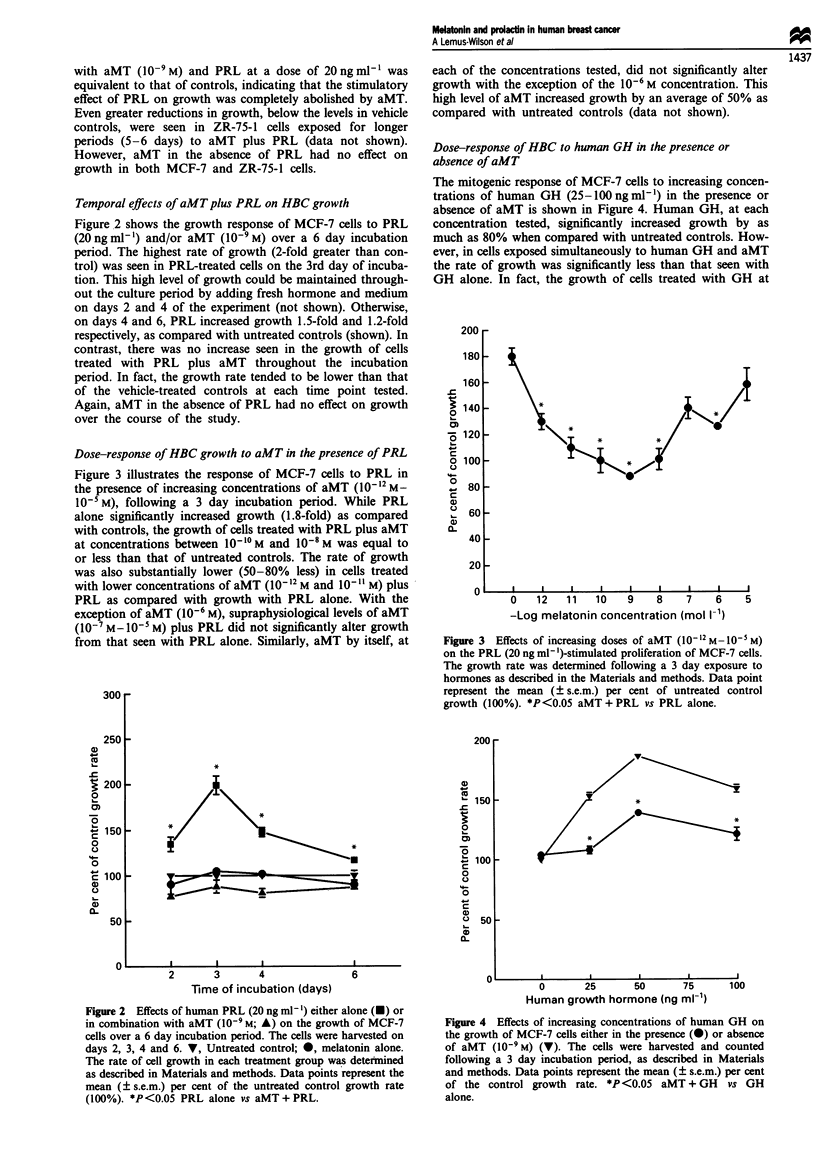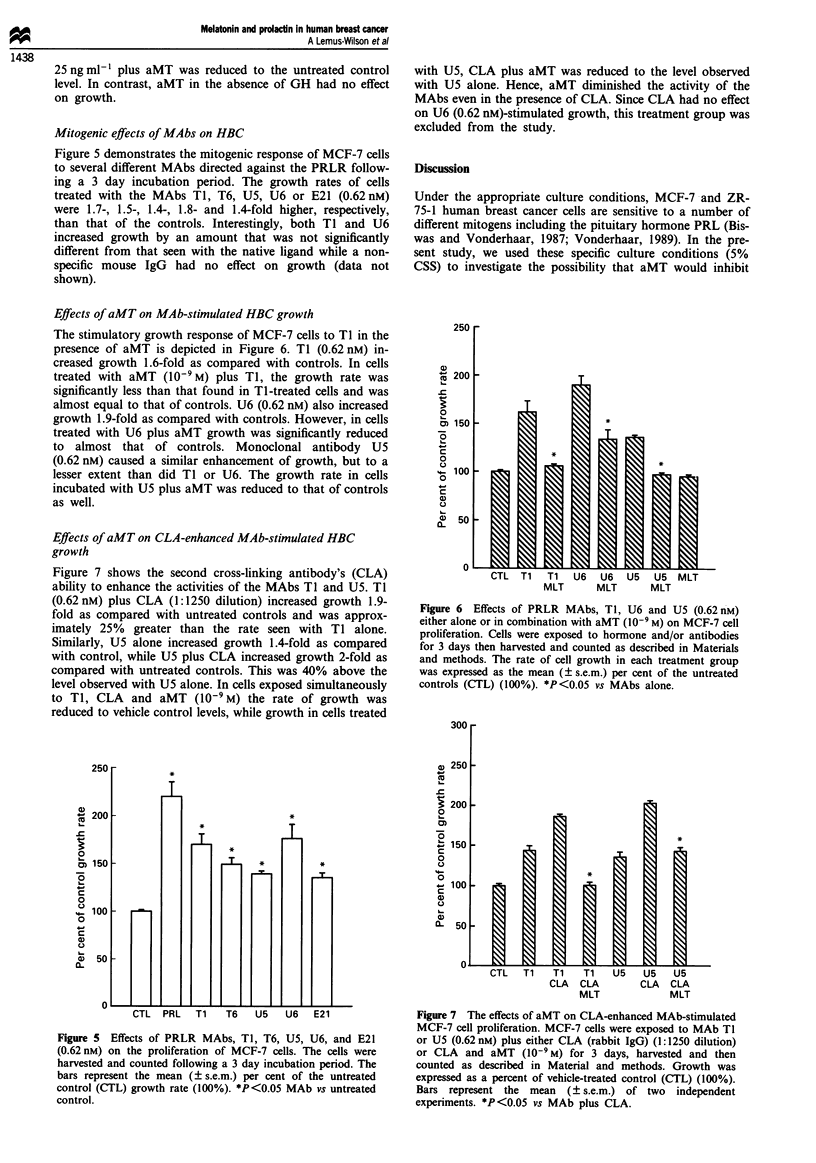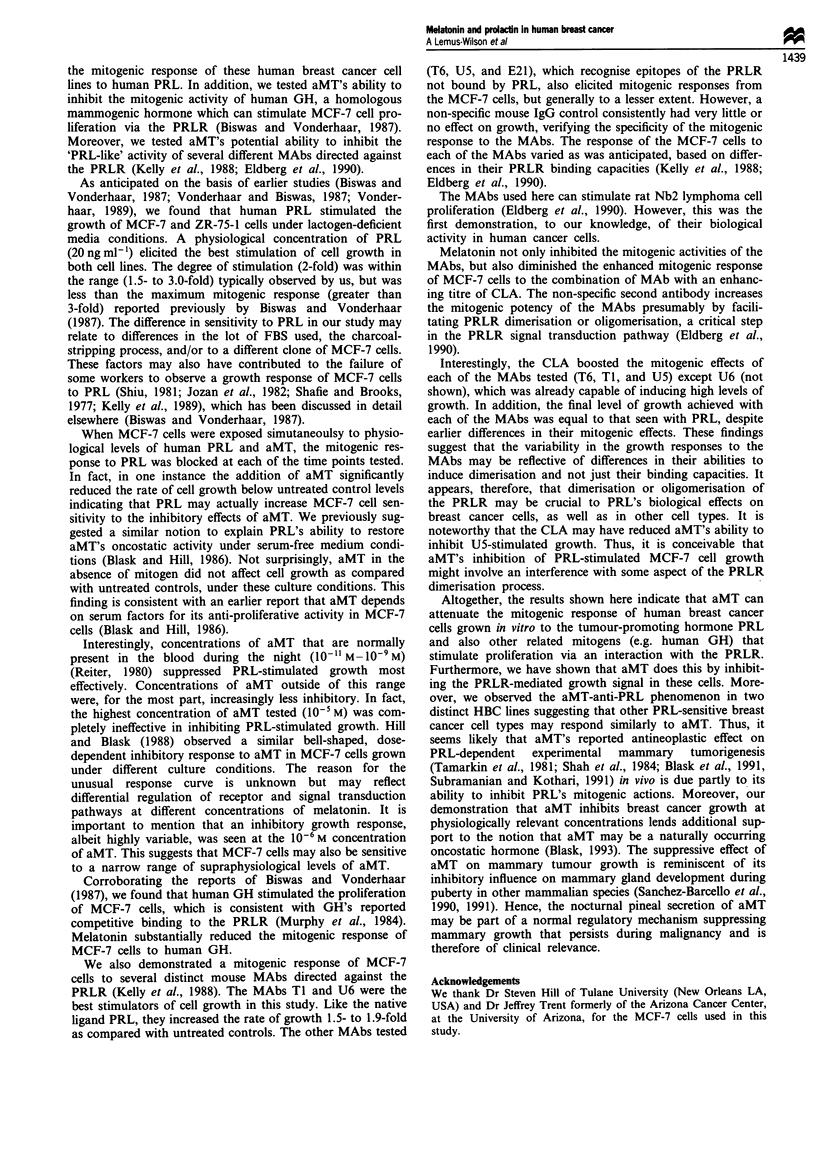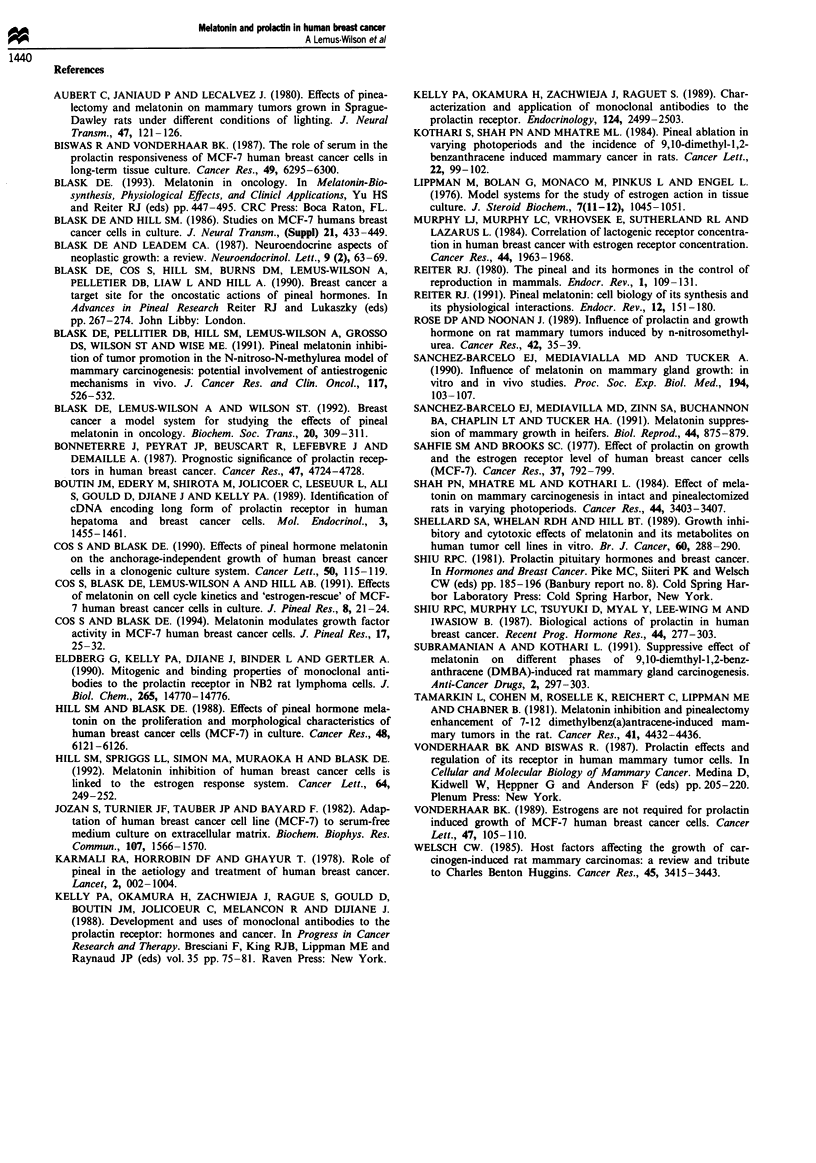# Melatonin blocks the stimulatory effects of prolactin on human breast cancer cell growth in culture.

**DOI:** 10.1038/bjc.1995.526

**Published:** 1995-12

**Authors:** A. Lemus-Wilson, P. A. Kelly, D. E. Blask

**Affiliations:** University of Arizona, Tucson, USA.

## Abstract

Melatonin (aMT) appears to be a potentially important oncostatic substance that can block the mitogenic effects of tumour-promoting hormones and growth factors such as oestradiol and epidermal growth factor, in vitro. In the present study, we examined the possibility that aMT would also inhibit the stimulatory effects of the tumour-promoter prolactin (PRL) on MCF-7 and ZR75-1 human breast cancer cell (HBC) growth under 5% charcoal-stripped fetal bovine serum culture conditions. Human PRL (10-100 ng ml-1) stimulated the rate of MCF-7 and ZR-75-1 HBC growth up to 2-fold above that of untreated controls. Melatonin, at concentrations between 10(-12) M and 10(-5)M, diminished and at physiological levels completely abolished PRL's mitogenic activity, but had no effect on growth in the absence of PRL. The mitogenic effects of human growth hormone (hGH), a PRL-related hormone, and also of several monoclonal antibodies (MAbs) against the PRL receptor (PRLR), were also abrogated by physiological concentrations of aMT. Additionally, aMT blocked the enhancement of MAb mitogenic activity induced by a second 'cross-linking' antibody (CLA). These findings indicate that aMT interrupts the PRLR-mediated growth signal in HBC and suggest that the oncostatic activity of aMT may also be linked with an antagonism of PRL's actions.


British Journal of Cancer (1995) 72, 1435-1440
© 1995 Stockton Press All rights reserved 0007-0920/95 $12.00

### Melatonin blocks the stimulatory effects of prolactin on human breast cancer cell growth in culture

A Lemus-Wilson[1,3], PA Kelly[2] and DE Blask[3]

[1]*The University of Arizona, Tucson, Arizona, USA;* [2]*INSERM Unité 344, Endocrinologie Moléculaire, Faculté de Médicine Necker, 75730 Paris, France;* [3]*The Bassett Research Institute, Cooperstown, New York, 13326-1394, USA.*

**Summary** Melatonin (aMT) appears to be a potentially important oncostatic substance that can block the mitogenic effects of tumour-promoting hormones and growth factors such as oestradiol and epidermal growth factor, *in vitro*. In the present study, we examined the possibility that aMT would also inhibit the stimulatory effects of the tumour-promoter prolactin (PRL) on MCF-7 and ZR75-1 human breast cancer cell (HBC) growth under 5% charcoal-stripped fetal bovine serum culture conditions. Human PRL (10-100 ng $ml^{-1}$) stimulated the rate of MCF-7 and ZR-75-1 HBC growth up to 2-fold above that of untreated controls. Melatonin, at concentrations between $10^{-12}$ M and $10^{-5}$ M, diminished and at physiological levels completely abolished PRL's mitogenic activity, but had no effect on growth in the absence of PRL. The mitogenic effects of human growth hormone (hGH), a PRL-related hormone, and also of several monoclonal antibodies (MAbs) against the PRL receptor (PRLR), were also abrogated by physiological concentrations of aMT. Additionally, aMT blocked the enhancement of MAb mitogenic activity induced by a second 'cross-linking' antibody (CLA). These findings indicate that aMT interrupts the PRLR-mediated growth signal in HBC and suggest that the oncostatic activity of aMT may also be linked with an antagonism of PRL's actions.

**Keywords:** melatonin; prolactin; human breast cancer

Melatonin (aMT), the chief hormone from the pineal gland (Reiter, 1991), appears to be an important inhibitor of cancer (Blask, 1993). Over the past decade a considerable amount of evidence has accumulated suggesting that aMT has potent antineoplastic properties, particularly with respect to the development of breast neoplasms (Blask *et al*., 1990, 1992). For example, several workers have shown that exogenously administered aMT impedes chemically induced mammary tumorigenesis in rodents (Aubert *et al*., 1980; Kothari *et al*., 1984; Blask *et al*., 1991). Moreover, the reduction of aMT in the blood, via surgical or functional pinealectomy, enhances carcinogenesis in susceptible animals but not those given aMT (Tamarkin *et al*., 1981; Shah *et al*., 1984). In addition, aMT impedes the growth of human breast cancer cells *in vitro* (Hill and Blask, 1988; Shellard *et al*., 1989; Cos and Blask, 1990) and breast tumours transplanted into rats (Karmali *et al*., 1978). Since these results suggest that circulating aMT in the blood may impart some protection against mammary tumorigenesis and that established tumours retain their sensitivity to aMT's cytostatic effects, it has been postulated that breast cancer may respond favourably to aMT therapy (Karmali *et al*., 1978). These ideas are also supported by aMT's ability to reduce the circulating levels and/or activity of mammogenic hormones such as oestrogen and prolactin (PRL) (Reiter, 1980; Blask and Leadem, 1987), both of which stimulate breast cancer growth (Welsch, 1985; Rose and Noonan, 1989). One of the most interesting aspects of aMT's anti-cancer activity is its ability to inhibit the growth of the tumour cell itself by blocking the stimulatory effects of other hormones and growth factors. For example, recent studies have shown that aMT can inhibit human breast cancer cell (HBC) proliferation by blocking the mitogenic actions of both oestradiol and epidermal growth factor (Cos *et al*., 1991; Hill *et al*., 1992; Cos and Blask, 1994), and causing a delay at the $G_0/G_1$ stage of the cell cycle (Cos *et al*., 1991).

In this study, we examined aMT's potential for blocking the mitogenic actions of PRL on HBCs grown in culture. The examination of aMT inhibition of PRL's mitogenic action, albeit not previously studied, is warranted, based on aMT's ability to inhibit PRL-dependent mammary tumours *in vivo* (Tamarkin *et al*., 1981; Shah *et al*., 1984; Blask *et al*., 1991; Subramanian and Kothari, 1991) and on the increasing concern that PRL promotes breast malignancies in humans (Bonneterre *et al*., 1987; Shiu *et al*., 1987; Boutin *et al*., 1989) as it does in rodents (Welsch, 1985). The effects of aMT on PRL-stimulated HBC growth were studied in the MCF-7 and ZR-75-1 oestrogen receptor (ER)-positive HBC models because of the widespread use of these cells and their established sensitivity to PRL under defined (lactogen-deficient) serum conditions (Biswas and Vonderhaar, 1987; Vonderhaar and Biswas, 1987; Vonderhaar, 1989).

We also tested aMT's ability to inhibit the mitogenic activity of a PRL-related hormone, human growth hormone (hGH), as well as the mitogenic effects of several mouse monoclonal antibodies (MAbs) directed against the PRL receptor (PRLR) (Kelly *et al*., 1988; Eldberg *et al*., 1990). The MAbs were tested by themselves and also in combination with a second antibody that boosts the mitogenic actions of the MAbs, resulting in a further increase in proliferation (Eldberg *et al*., 1990). These additional studies helped clarify aMT's anti-PRL action since both hGH (Biswas and Vonderhaar, 1987) and the MAbs (Kelly *et al*., 1988) obstensibly stimulate proliferation through the PRLR.

### Materials and methods

### *Human breast cancer cells*

Human MCF-7 breast cancer cells were obtained from Dr Steven Hill of Tulane University (New Orleans, LA, USA), and also from American Type Culture Collection, Rockville, MD, USA. The ZR-75-1 breast cancer cells were purchased from the American Type Culture Collection. All cells were routinely tested for mycoplasma contamination and were determined to be mycoplasma free.

### *Other materials and hormones*

Culture media, antibiotics and trypan blue were obtained from Gibco (Grand Island, NY, USA). Fetal bovine serum was obtained from Gibco and Tissue Culture Biologicals (Tulare, CA, USA). Dextran T-70 was supplied by Pharmacia (Piscataway, NJ, USA). Melatonin was obtained from

Correspondence: DE Blask, The Bassett Research Institute, (607) 547-3677, Cooperstown, New York, 13326-1394, USA
Received 19 April 1995; revised 28 June 1995; accepted 7 July 1995

Sigma (St Louis, MO, USA). Human PRL (NIADDK-hPRL-1-6, >99.9% purity) and Human GH (NIDDK-hGH-B-1) were generous gifts from Dr Salvatore Raiti of the National Pituitary Hormone Program. Monoclonal antibodies to the PRLR were those described previously (Kelly *et al.*, 1988, 1989) that have been shown to cross-react with the human PRLR (Eldberg *et al.*, 1990). Rabbit antiserum to mouse immunoglobulin G was purchased from Calbiochem (La Jolla, CA, USA).

### *Culture techniques*

MCF-7 and ZR-75-1 cells were maintained and routinely subpassaged in Dulbecco's modified Eagle medium (DMEM) supplemented with penicillin (200 U $ml^{-1}$), streptomycin (200 μg $ml^{-1}$), and 10% fetal bovine serum (FBS), at 37°C in a humid atmosphere containing 5% carbon dioxide. Before growth studies, cells were grown for at least 4 days in 10% charcoal-stripped FBS (CSS) to minimise the carry-over of bovine lactogens into the experiments.

### *Preparation of charcoal-stripped serum*

A modification of the charcoal-stripping methods of Lippman *et al.* (1976) was used in the present study. Briefly, Norit A charcoal was repeatedly washed first with 1 M hydrogen chloride then with phosphate-buffered saline (PBS) containing 1 mg $ml^{-1}$ of Dextran T-70 pH 7.2. The charcoal suspension was then dried in an oven overnight and 25 mg of dextran-coated charcoal was added to 500 ml of FBS at 56°C for 30 min. This suspension was then centrifuged at 10 000 *g* for 45 min and prefiltered through a millipore glass fibre filter. The serum was then re-exposed to dextran-coated charcoal for 1–2 additional cycles in order to further reduce its lactogen content. The CSS was sterilised by passage through a 0.22 μM millipore filter and then stored at −20°C until needed. This procedure reduced the bovine lactogen content of the serum by 98% (<3 ng $ml^{-1}$) as determined by radioimmunoassay (RIA). In addition, 83% of the endogenous oestrogens were removed as determined by RIA.

### In vitro *cell growth studies*

Cells were harvested from 70–80% confluent cultures and reseeded onto 60 mm × 15 mm Falcon plates at a density of $3 \times 10^5$ cells/plate. DMEM supplemented with 5% lactogen-deficient CSS and 2% antibiotics was used for the culture of both MCF-7 and ZR-75-1 cells. After 24 h the medium was replaced with fresh DMEM containing either vehicle, hormones and/or MAbs. Melatonin was dissolved in ethanol (EtOH) then diluted with medium to $10^{-12}$–$10^{-5}$ M. The level of EtOH carried over into the studies was not more than 0.035% (vehicle control). The cells were allowed to grow for up to 6 days in this medium. Cell number for triplicate plates for each treatment was determined with a haemocytometer after detachment of the cells with PBS/EDTA and their passage through a 25 gauge syringe. Cell viability (>95%) was assessed by the trypan blue exclusion test. A mean growth rate from triplicate plates was calculated based on cell number. The growth rate was equal to the difference between the initial number of cells seeded per plate and the number of cells found at a particular time point, at any time from 24–144 h, divided by the initial cell number. For comparisons between groups the mean rate of growth in each treatment group was graphed as a per cent of control growth rate. Unless otherwise stated the data are expressed as the mean of three independent experiments.

For the PRL dose–response studies 20–100 ng $ml^{-1}$ of hormone was used, the other experiments used only the 20 ng $ml^{-1}$ dose. In the hGH dose–response studies 20–100 ng $ml^{-1}$ of hormone was used. In the MAb studies, MAbs T1, T6, U5, U6 or E21 were each used at a final concentration of 0.62 nM. In the experiments with the CLA, a dilution of 1:1250 was used because it was the most effective at enhancing the mitogenic activities of the T1 and T6 MAbs.

### *Statistical analysis*

Mean ± (s.e.m.) growth rate for hormone-treated cells was expressed as a per cent of the control growth rate (100%). All data were analysed with a one-way ANOVA and statistical differences among mean values were determined by Student Newman–Keuls *post hoc* test. Differences between and among means were considered significant at $P<0.05$.

### Results

### *Dose–response of HBC growth to PRL in the presence or absence of aMT*

As shown in Figure 1a and b, PRL, at concentrations ranging from 20 to 100 ng $ml^{-1}$, significantly increased the rate of MCF-7 and ZR-75-1 cell growth as compared with untreated controls when cells were grown for 3 days as monolayer cultures in medium supplemented with 5% CSS and hormone. A physiological dose of PRL (20 ng $ml^{-1}$) caused the greatest increase (2-fold) in both MCF-7 and ZR-75-1 cell growth as compared with untreated control.

Melatonin ($10^{-9}$ M) substantially reduced the growth of both MCF-7 and ZR-75-1 cells in the presence of increasing doses of PRL (Figure 1a and b). In fact, the rate of growth in both MCF-7 cell and ZR-75-1 cells treated concomitantly

**Figure 1** Effects of aMT on the human PRL-stimulated growth of MCF-7 (**a**) and ZR-75-1 (**b**) human breast cancer cells. Cells were treated with PRL at concentrations ranging from 20 to 100 ng $ml^{-1}$ in the presence of aMT ($10^{-9}$ M) (O) or in the absence of aMT (∇). The growth rate was determined as described in Materials and methods following a 3 day exposure to hormones. The data points represent the mean (± s.e.m.) per cent of untreated control growth rate (100%) from three separate experiments. aMT + PRL *vs* PRL $P<0.05$ at all concentrations of PRL.

with aMT ($10^{-9}$ M) and PRL at a dose of $20\,ng\,ml^{-1}$ was equivalent to that of controls, indicating that the stimulatory effect of PRL on growth was completely abolished by aMT. Even greater reductions in growth, below the levels in vehicle controls, were seen in ZR-75-1 cells exposed for longer periods (5–6 days) to aMT plus PRL (data not shown). However, aMT in the absence of PRL had no effect on growth in both MCF-7 and ZR-75-1 cells.

*Temporal effects of aMT plus PRL on HBC growth*

Figure 2 shows the growth response of MCF-7 cells to PRL ($20\,ng\,ml^{-1}$) and/or aMT ($10^{-9}$ M) over a 6 day incubation period. The highest rate of growth (2-fold greater than control) was seen in PRL-treated cells on the 3rd day of incubation. This high level of growth could be maintained throughout the culture period by adding fresh hormone and medium on days 2 and 4 of the experiment (not shown). Otherwise, on days 4 and 6, PRL increased growth 1.5-fold and 1.2-fold respectively, as compared with untreated controls (shown). In contrast, there was no increase seen in the growth of cells treated with PRL plus aMT throughout the incubation period. In fact, the growth rate tended to be lower than that of the vehicle-treated controls at each time point tested. Again, aMT in the absence of PRL had no effect on growth over the course of the study.

*Dose–response of HBC growth to aMT in the presence of PRL*

Figure 3 illustrates the response of MCF-7 cells to PRL in the presence of increasing concentrations of aMT ($10^{-12}$ M–$10^{-5}$ M), following a 3 day incubation period. While PRL alone significantly increased growth (1.8-fold) as compared with controls, the growth of cells treated with PRL plus aMT at concentrations between $10^{-10}$ M and $10^{-8}$ M was equal to or less than that of untreated controls. The rate of growth was also substantially lower (50–80% less) in cells treated with lower concentrations of aMT ($10^{-12}$ M and $10^{-11}$ M) plus PRL as compared with growth with PRL alone. With the exception of aMT ($10^{-6}$ M), supraphysiological levels of aMT ($10^{-7}$ M–$10^{-5}$ M) plus PRL did not significantly alter growth from that seen with PRL alone. Similarly, aMT by itself, at each of the concentrations tested, did not significantly alter growth with the exception of the $10^{-6}$ M concentration. This high level of aMT increased growth by an average of 50% as compared with untreated controls (data not shown).

*Dose–response of HBC to human GH in the presence or absence of aMT*

The mitogenic response of MCF-7 cells to increasing concentrations of human GH (25–100 $ng\,ml^{-1}$) in the presence or absence of aMT is shown in Figure 4. Human GH, at each concentration tested, significantly increased growth by as much as 80% when compared with untreated controls. However, in cells exposed simultaneously to human GH and aMT the rate of growth was significantly less than that seen with GH alone. In fact, the growth of cells treated with GH at

**Figure 2** Effects of human PRL ($20\,ng\,ml^{-1}$) either alone (■) or in combination with aMT ($10^{-9}$ M; ▲) on the growth of MCF-7 cells over a 6 day incubation period. The cells were harvested on days 2, 3, 4 and 6. ▼, Untreated control; ●, melatonin alone. The rate of cell growth in each treatment group was determined as described in Materials and methods. Data points represent the mean (± s.e.m.) per cent of the untreated control growth rate (100%). *$P<0.05$ PRL alone *vs* aMT + PRL.

**Figure 3** Effects of increasing doses of aMT ($10^{-12}$ M–$10^{-5}$ M) on the PRL ($20\,ng\,ml^{-1}$)-stimulated proliferation of MCF-7 cells. The growth rate was determined following a 3 day exposure to hormones as described in the Materials and methods. Data point represent the mean (± s.e.m.) per cent of untreated control growth (100%). *$P<0.05$ aMT + PRL *vs* PRL alone.

**Figure 4** Effects of increasing concentrations of human GH on the growth of MCF-7 cells either in the presence (●) or absence of aMT ($10^{-9}$ M) (▼). The cells were harvested and counted following a 3 day incubation period, as described in Materials and methods. Data points represent the mean (± s.e.m.) per cent of the control growth rate. *$P<0.05$ aMT + GH *vs* GH alone.

25 ng ml$^{-1}$ plus aMT was reduced to the untreated control level. In contrast, aMT in the absence of GH had no effect on growth.

### *Mitogenic effects of MAbs on HBC*

Figure 5 demonstrates the mitogenic response of MCF-7 cells to several different MAbs directed against the PRLR following a 3 day incubation period. The growth rates of cells treated with the MAbs T1, T6, U5, U6 or E21 (0.62 nM) were 1.7-, 1.5-, 1.4-, 1.8- and 1.4-fold higher, respectively, than that of the controls. Interestingly, both T1 and U6 increased growth by an amount that was not significantly different from that seen with the native ligand while a non-specific mouse IgG had no effect on growth (data not shown).

### *Effects of aMT on MAb-stimulated HBC growth*

The stimulatory growth response of MCF-7 cells to T1 in the presence of aMT is depicted in Figure 6. T1 (0.62 nM) increased growth 1.6-fold as compared with controls. In cells treated with aMT ($10^{-9}$ M) plus T1, the growth rate was significantly less than that found in T1-treated cells and was almost equal to that of controls. U6 (0.62 nM) also increased growth 1.9-fold as compared with controls. However, in cells treated with U6 plus aMT growth was significantly reduced to almost that of controls. Monoclonal antibody U5 (0.62 nM) caused a similar enhancement of growth, but to a lesser extent than did T1 or U6. The growth rate in cells incubated with U5 plus aMT was reduced to that of controls as well.

### *Effects of aMT on CLA-enhanced MAb-stimulated HBC growth*

Figure 7 shows the second cross-linking antibody's (CLA) ability to enhance the activities of the MAbs T1 and U5. T1 (0.62 nM) plus CLA (1:1250 dilution) increased growth 1.9-fold as compared with untreated controls and was approximately 25% greater than the rate seen with T1 alone. Similarly, U5 alone increased growth 1.4-fold as compared with control, while U5 plus CLA increased growth 2-fold as compared with untreated controls. This was 40% above the level observed with U5 alone. In cells exposed simultaneously to T1, CLA and aMT ($10^{-9}$ M) the rate of growth was reduced to vehicle control levels, while growth in cells treated with U5, CLA plus aMT was reduced to the level observed with U5 alone. Hence, aMT diminished the activity of the MAbs even in the presence of CLA. Since CLA had no effect on U6 (0.62 nM)-stimulated growth, this treatment group was excluded from the study.

### Discussion

Under the appropriate culture conditions, MCF-7 and ZR-75-1 human breast cancer cells are sensitive to a number of different mitogens including the pituitary hormone PRL (Biswas and Vonderhaar, 1987; Vonderhaar, 1989). In the present study, we used these specific culture conditions (5% CSS) to investigate the possibility that aMT would inhibit

**Figure 5** Effects of PRLR MAbs, T1, T6, U5, U6, and E21 (0.62 nM) on the proliferation of MCF-7 cells. The cells were harvested and counted following a 3 day incubation period. The bars represent the mean (± s.e.m.) per cent of the untreated control (CTL) growth rate (100%). *$P$<0.05 MAb *vs* untreated control.

**Figure 6** Effects of PRLR MAbs, T1, U6 and U5 (0.62 nM) either alone or in combination with aMT ($10^{-9}$ M) on MCF-7 cell proliferation. Cells were exposed to hormone and/or antibodies for 3 days then harvested and counted as described in Materials and methods. The rate of cell growth in each treatment group was expressed as the mean (± s.e.m.) per cent of the untreated controls (CTL) (100%). *$P$<0.05 *vs* MAbs alone.

**Figure 7** The effects of aMT on CLA-enhanced MAb-stimulated MCF-7 cell proliferation. MCF-7 cells were exposed to MAb T1 or U5 (0.62 nM) plus either CLA (rabbit IgG) (1:1250 dilution) or CLA and aMT ($10^{-9}$ M) for 3 days, harvested and then counted as described in Material and methods. Growth was expressed as a percent of vehicle-treated control (CTL) (100%). Bars represent the mean (± s.e.m.) of two independent experiments. *$P$<0.05 *vs* MAb plus CLA.

the mitogenic response of these human breast cancer cell lines to human PRL. In addition, we tested aMT's ability to inhibit the mitogenic activity of human GH, a homologous mammogenic hormone which can stimulate MCF-7 cell proliferation via the PRLR (Biswas and Vonderhaar, 1987). Moreover, we tested aMT's potential ability to inhibit the 'PRL-like' activity of several different MAbs directed against the PRLR (Kelly *et al.*, 1988; Eldberg *et al.*, 1990).

As anticipated on the basis of earlier studies (Biswas and Vonderhaar, 1987; Vonderhaar and Biswas, 1987; Vonderhaar, 1989), we found that human PRL stimulated the growth of MCF-7 and ZR-75-1 cells under lactogen-deficient media conditions. A physiological concentration of PRL ($20\ ng\ ml^{-1}$) elicited the best stimulation of cell growth in both cell lines. The degree of stimulation (2-fold) was within the range (1.5- to 3.0-fold) typically observed by us, but was less than the maximum mitogenic response (greater than 3-fold) reported previously by Biswas and Vonderhaar (1987). The difference in sensitivity to PRL in our study may relate to differences in the lot of FBS used, the charcoal-stripping process, and/or to a different clone of MCF-7 cells. These factors may also have contributed to the failure of some workers to observe a growth response of MCF-7 cells to PRL (Shiu, 1981; Jozan *et al.*, 1982; Shafie and Brooks, 1977; Kelly *et al.*, 1989), which has been discussed in detail elsewhere (Biswas and Vonderhaar, 1987).

When MCF-7 cells were exposed simutaneoulsy to physiological levels of human PRL and aMT, the mitogenic response to PRL was blocked at each of the time points tested. In fact, in one instance the addition of aMT significantly reduced the rate of cell growth below untreated control levels indicating that PRL may actually increase MCF-7 cell sensitivity to the inhibitory effects of aMT. We previously suggested a similar notion to explain PRL's ability to restore aMT's oncostatic activity under serum-free medium conditions (Blask and Hill, 1986). Not surprisingly, aMT in the absence of mitogen did not affect cell growth as compared with untreated controls, under these culture conditions. This finding is consistent with an earlier report that aMT depends on serum factors for its anti-proliferative activity in MCF-7 cells (Blask and Hill, 1986).

Interestingly, concentrations of aMT that are normally present in the blood during the night ($10^{-11}\ M-10^{-9}\ M$) (Reiter, 1980) suppressed PRL-stimulated growth most effectively. Concentrations of aMT outside of this range were, for the most part, increasingly less inhibitory. In fact, the highest concentration of aMT tested ($10^{-5}\ M$) was completely ineffective in inhibiting PRL-stimulated growth. Hill and Blask (1988) observed a similar bell-shaped, dose-dependent inhibitory response to aMT in MCF-7 cells grown under different culture conditions. The reason for the unusual response curve is unknown but may reflect differential regulation of receptor and signal transduction pathways at different concentrations of melatonin. It is important to mention that an inhibitory growth response, albeit highly variable, was seen at the $10^{-6}\ M$ concentration of aMT. This suggests that MCF-7 cells may also be sensitive to a narrow range of supraphysiological levels of aMT.

Corroborating the reports of Biswas and Vonderhaar (1987), we found that human GH stimulated the proliferation of MCF-7 cells, which is consistent with GH's reported competitive binding to the PRLR (Murphy *et al.*, 1984). Melatonin substantially reduced the mitogenic response of MCF-7 cells to human GH.

We also demonstrated a mitogenic response of MCF-7 cells to several distinct mouse MAbs directed against the PRLR (Kelly *et al.*, 1988). The MAbs T1 and U6 were the best stimulators of cell growth in this study. Like the native ligand PRL, they increased the rate of growth 1.5- to 1.9-fold as compared with untreated controls. The other MAbs tested (T6, U5, and E21), which recognise epitopes of the PRLR not bound by PRL, also elicited mitogenic responses from the MCF-7 cells, but generally to a lesser extent. However, a non-specific mouse IgG control consistently had very little or no effect on growth, verifying the specificity of the mitogenic response to the MAbs. The response of the MCF-7 cells to each of the MAbs varied as was anticipated, based on differences in their PRLR binding capacities (Kelly *et al.*, 1988; Eldberg *et al.*, 1990).

The MAbs used here can stimulate rat Nb2 lymphoma cell proliferation (Eldberg *et al.*, 1990). However, this was the first demonstration, to our knowledge, of their biological activity in human cancer cells.

Melatonin not only inhibited the mitogenic activities of the MAbs, but also diminished the enhanced mitogenic response of MCF-7 cells to the combination of MAb with an enhancing titre of CLA. The non-specific second antibody increases the mitogenic potency of the MAbs presumably by facilitating PRLR dimerisation or oligomerisation, a critical step in the PRLR signal transduction pathway (Eldberg *et al.*, 1990).

Interestingly, the CLA boosted the mitogenic effects of each of the MAbs tested (T6, T1, and U5) except U6 (not shown), which was already capable of inducing high levels of growth. In addition, the final level of growth achieved with each of the MAbs was equal to that seen with PRL, despite earlier differences in their mitogenic effects. These findings suggest that the variability in the growth responses to the MAbs may be reflective of differences in their abilities to induce dimerisation and not just their binding capacities. It appears, therefore, that dimerisation or oligomerisation of the PRLR may be crucial to PRL's biological effects on breast cancer cells, as well as in other cell types. It is noteworthy that the CLA may have reduced aMT's ability to inhibit U5-stimulated growth. Thus, it is conceivable that aMT's inhibition of PRL-stimulated MCF-7 cell growth might involve an interference with some aspect of the PRLR dimerisation process.

Altogether, the results shown here indicate that aMT can attenuate the mitogenic response of human breast cancer cells grown *in vitro* to the tumour-promoting hormone PRL and also other related mitogens (e.g. human GH) that stimulate proliferation via an interaction with the PRLR. Furthermore, we have shown that aMT does this by inhibiting the PRLR-mediated growth signal in these cells. Moreover, we observed the aMT-anti-PRL phenomenon in two distinct HBC lines suggesting that other PRL-sensitive breast cancer cell types may respond similarly to aMT. Thus, it seems likely that aMT's reported antineoplastic effect on PRL-dependent experimental mammary tumorigenesis (Tamarkin *et al.*, 1981; Shah *et al.*, 1984; Blask *et al.*, 1991, Subramanian and Kothari, 1991) *in vivo* is due partly to its ability to inhibit PRL's mitogenic actions. Moreover, our demonstration that aMT inhibits breast cancer growth at physiologically relevant concentrations lends additional support to the notion that aMT may be a naturally occurring oncostatic hormone (Blask, 1993). The suppressive effect of aMT on mammary tumour growth is reminiscent of its inhibitory influence on mammary gland development during puberty in other mammalian species (Sanchez-Barcello *et al.*, 1990, 1991). Hence, the nocturnal pineal secretion of aMT may be part of a normal regulatory mechanism suppressing mammary growth that persists during malignancy and is therefore of clinical relevance.

**Acknowledgements**
We thank Dr Steven Hill of Tulane University (New Orleans LA, USA) and Dr Jeffrey Trent formerly of the Arizona Cancer Center, at the University of Arizona, for the MCF-7 cells used in this study.

### References

AUBERT C, JANIAUD P AND LECALVEZ J. (1980). Effects of pinealectomy and melatonin on mammary tumors grown in Sprague-Dawley rats under different conditions of lighting. *J. Neural Transm.*, **47**, 121–126.

BISWAS R AND VONDERHAAR BK. (1987). The role of serum in the prolactin responsiveness of MCF-7 human breast cancer cells in long-term tissue culture. *Cancer Res.*, **49**, 6295–6300.

BLASK DE. (1993). Melatonin in oncology. In *Melatonin-Biosynthesis, Physiological Effects, and Clinicl Applications*, Yu HS and Reiter RJ (eds) pp. 447–495. CRC Press: Boca Raton, FL.

BLASK DE AND HILL SM. (1986). Studies on MCF-7 humans breast cancer cells in culture. *J. Neural Transm.*, **(Suppl) 21**, 433–449.

BLASK DE AND LEADEM CA. (1987). Neuroendocrine aspects of neoplastic growth: a review. *Neuroendocrinol. Lett.*, **9 (2)**, 63–69.

BLASK DE, COS S, HILL SM, BURNS DM, LEMUS-WILSON A, PELLETIER DB, LIAW L AND HILL A. (1990). Breast cancer a target site for the oncostatic actions of pineal hormones. In *Advances in Pineal Research* Reiter RJ and Lukaszky (eds) pp. 267–274. John Libby: London.

BLASK DE, PELLITIER DB, HILL SM, LEMUS-WILSON A, GROSSO DS, WILSON ST AND WISE ME. (1991). Pineal melatonin inhibition of tumor promotion in the N-nitroso-N-methylurea model of mammary carcinogenesis: potential involvement of antiestrogenic mechanisms in vivo. *J. Cancer Res. and Clin. Oncol.*, **117**, 526–532.

BLASK DE, LEMUS-WILSON A AND WILSON ST. (1992). Breast cancer a model system for studying the effects of pineal melatonin in oncology. *Biochem. Soc. Trans.*, **20**, 309–311.

BONNETERRE J, PEYRAT JP, BEUSCART R, LEFEBVRE J AND DEMAILLE A. (1987). Prognostic significance of prolactin receptors in human breast cancer. *Cancer Res.*, **47**, 4724–4728.

BOUTIN JM, EDERY M, SHIROTA M, JOLICOER C, LESEUUR L, ALI S, GOULD D, DJIANE J AND KELLY PA. (1989). Identification of cDNA encoding long form of prolactin receptor in human hepatoma and breast cancer cells. *Mol. Endocrinol.*, **3**, 1455–1461.

COS S AND BLASK DE. (1990). Effects of pineal hormone melatonin on the anchorage-independent growth of human breast cancer cells in a clonogenic culture system. *Cancer Lett.*, **50**, 115–119.

COS S, BLASK DE, LEMUS-WILSON A AND HILL AB. (1991). Effects of melatonin on cell cycle kinetics and 'estrogen-rescue' of MCF-7 human breast cancer cells in culture. *J. Pineal Res.*, **8**, 21–24.

COS S AND BLASK DE. (1994). Melatonin modulates growth factor activity in MCF-7 human breast cancer cells. *J. Pineal Res.*, **17**, 25–32.

ELDBERG G, KELLY PA, DJIANE J, BINDER L AND GERTLER A. (1990). Mitogenic and binding properties of monoclonal antibodies to the prolactin receptor in NB2 rat lymphoma cells. *J. Biol. Chem.*, **265**, 14770–14776.

HILL SM AND BLASK DE. (1988). Effects of pineal hormone melatonin on the proliferation and morphological characteristics of human breast cancer cells (MCF-7) in culture. *Cancer Res.*, **48**, 6121–6126.

HILL SM, SPRIGGS LL, SIMON MA, MURAOKA H AND BLASK DE. (1992). Melatonin inhibition of human breast cancer cells is linked to the estrogen response system. *Cancer Lett.*, **64**, 249–252.

JOZAN S, TURNIER JF, TAUBER JP AND BAYARD F. (1982). Adaptation of human breast cancer cell line (MCF-7) to serum-free medium culture on extracellular matrix. *Biochem. Biophys. Res. Commun.*, **107**, 1566–1570.

KARMALI RA, HORROBIN DF AND GHAYUR T. (1978). Role of pineal in the aetiology and treatment of human breast cancer. *Lancet*, **2**, 002–1004.

KELLY PA, OKAMURA H, ZACHWIEJA J, RAGUE S, GOULD D, BOUTIN JM, JOLICOEUR C, MELANCON R AND DIJIANE J. (1988). Development and uses of monoclonal antibodies to the prolactin receptor: hormones and cancer. In *Progress in Cancer Research and Therapy*. Bresciani F, King RJB, Lippman ME and Raynaud JP (eds) vol. 35 pp. 75–81. Raven Press: New York.

KELLY PA, OKAMURA H, ZACHWIEJA J, RAGUET S. (1989). Characterization and application of monoclonal antibodies to the prolactin receptor. *Endocrinology*, **124**, 2499–2503.

KOTHARI S, SHAH PN AND MHATRE ML. (1984). Pineal ablation in varying photoperiods and the incidence of 9,10-dimethyl-1,2-benzanthracene induced mammary cancer in rats. *Cancer Lett.*, **22**, 99–102.

LIPPMAN M, BOLAN G, MONACO M, PINKUS L AND ENGEL L. (1976). Model systems for the study of estrogen action in tissue culture. *J. Steroid Biochem.*, **7(11–12)**, 1045–1051.

MURPHY LJ, MURPHY LC, VRHOVSEK E, SUTHERLAND RL AND LAZARUS L. (1984). Correlation of lactogenic receptor concentration in human breast cancer with estrogen receptor concentration. *Cancer Res.*, **44**, 1963–1968.

REITER RJ. (1980). The pineal and its hormones in the control of reproduction in mammals. *Endocr. Rev.*, **1**, 109–131.

REITER RJ. (1991). Pineal melatonin: cell biology of its synthesis and its physiological interactions. *Endocr. Rev.*, **12**, 151–180.

ROSE DP AND NOONAN J. (1989). Influence of prolactin and growth hormone on rat mammary tumors induced by n-nitrosomethylurea. *Cancer Res.*, **42**, 35–39.

SANCHEZ-BARCELO EJ, MEDIAVIALLA MD AND TUCKER A. (1990). Influence of melatonin on mammary gland growth: in vitro and in vivo studies. *Proc. Soc. Exp. Biol. Med.*, **194**, 103–107.

SANCHEZ-BARCELO EJ, MEDIAVILLA MD, ZINN SA, BUCHANNON BA, CHAPLIN LT AND TUCKER HA. (1991). Melatonin suppression of mammary growth in heifers. *Biol. Reprod.*, **44**, 875–879.

SAHFIE SM AND BROOKS SC. (1977). Effect of prolactin on growth and the estrogen receptor level of human breast cancer cells (MCF-7). *Cancer Res.*, **37**, 792–799.

SHAH PN, MHATRE ML AND KOTHARI L. (1984). Effect of melatonin on mammary carcinogenesis in intact and pinealectomized rats in varying photoperiods. *Cancer Res.*, **44**, 3403–3407.

SHELLARD SA, WHELAN RDH AND HILL BT. (1989). Growth inhibitory and cytotoxic effects of melatonin and its metabolites on human tumor cell lines in vitro. *Br. J. Cancer*, **60**, 288–290.

SHIU RPC. (1981). Prolactin pituitary hormones and breast cancer. In *Hormones and Breast Cancer*. Pike MC, Siiteri PK and Welsch CW (eds) pp. 185–196 (Banbury report no. 8). Cold Spring Harbor Laboratory Press: Cold Spring Harbor, New York.

SHIU RPC, MURPHY LC, TSUYUKI D, MYAL Y, LEE-WING M AND IWASIOW B. (1987). Biological actions of prolactin in human breast cancer. *Recent Prog. Hormone Res.*, **44**, 277–303.

SUBRAMANIAN A AND KOTHARI L. (1991). Suppressive effect of melatonin on different phases of 9,10-diemthyl-1,2-benzanthracene (DMBA)-induced rat mammary gland carcinogenesis. *Anti-Cancer Drugs*, **2**, 297–303.

TAMARKIN L, COHEN M, ROSELLE K, REICHERT C, LIPPMAN ME AND CHABNER B. (1981). Melatonin inhibition and pinealectomy enhancement of 7-12 dimethylbenz(a)antracene-induced mammary tumors in the rat. *Cancer Res.*, **41**, 4432–4436.

VONDERHAAR BK AND BISWAS R. (1987). Prolactin effects and regulation of its receptor in human mammary tumor cells. In *Cellular and Molecular Biology of Mammary Cancer*. Medina D, Kidwell W, Heppner G and Anderson F (eds) pp. 205–220. Plenum Press: New York.

VONDERHAAR BK. (1989). Estrogens are not required for prolactin induced growth of MCF-7 human breast cancer cells. *Cancer Lett.*, **47**, 105–110.

WELSCH CW. (1985). Host factors affecting the growth of carcinogen-induced rat mammary carcinomas: a review and tribute to Charles Benton Huggins. *Cancer Res.*, **45**, 3415–3443.